# Cutaneous tuberculosis chancre: case description in a child^☆^

**DOI:** 10.1016/j.abd.2020.12.016

**Published:** 2022-07-16

**Authors:** Gabriela Roncada Haddad, Lívia Caramaschi Florêncio, Vidal Haddad Junior

**Affiliations:** Department of Infectology, Dermatology, Diagnostic Imaging and Radiotherapy, Faculty of Medicine, Universidade Estadual Paulista, Botucatu, SP, Brazil

Dear Editor,

Cutaneous tuberculosis (CTB) is an infection caused by *Mycobacterium tuberculosis*, *M. bovis* or Bacillus Calmette-Guérin (BCG), used in immunizations. Clinical manifestations are variable and depend on several factors, such as the host’s immune status.^1^, ^2^

The extrapulmonary forms of tuberculosis account for approximately 10% of cases, with 1% to 2% occurring on the skin.^3^, ^4^ Children have this form of disease more frequently, possibly due to the immaturity of their immune system.

An eight-year-old male patient presented with an erythematous papule on the medial aspect of the right thigh, which developed into an ulcerated nodule followed by the appearance of another ulcerated nodule nearby after a few days (Fig. 1). He denied local trauma or systemic symptoms. On dermatological examination, there was an indurated plaque with 2 well-defined lesions (measuring 3 × 2 cm and 2 × 1.5 cm), with a fibrin base and brownish edges, painful on palpation. The inguinal lymph nodes were palpable bilaterally. Ultrasound examination showed a mass immediately below the lesion, with lobulated contours and low vascular flow, extending to the deep adipose plane. The lymph nodes were enlarged, showing regular contours and loss of the usual architecture.

**Figure 1:**
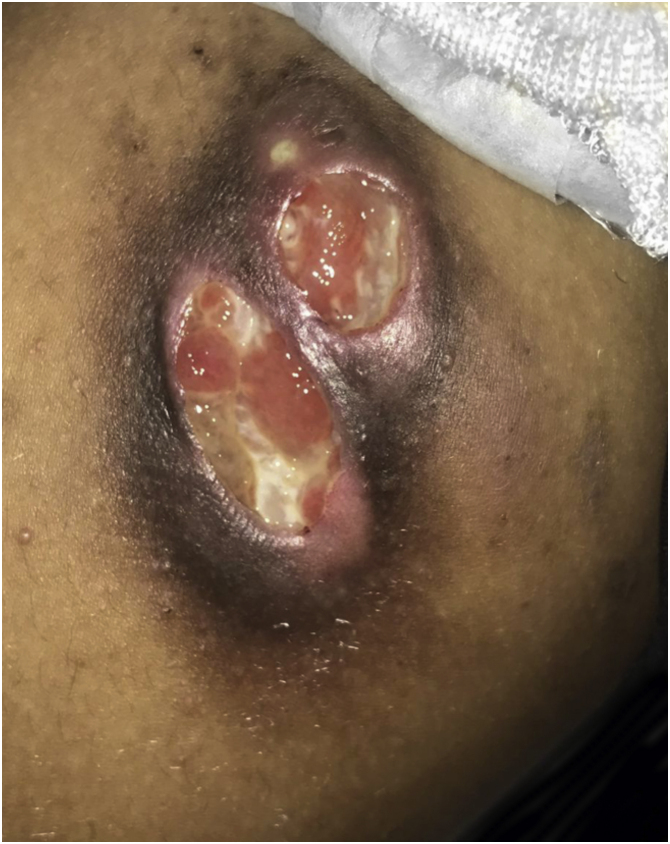
Ulcerated nodules on the medial aspect of the right thigh.

A wedge biopsy of the larger lesion was performed, which showed a granulomatous inflammatory process with areas of caseous necrosis, lymphohistiocytic inflammatory infiltrate, and Langerhans-type giant cells, favoring the diagnosis of tuberculosis.

The fungal screening tests (Grocott-Gomori and periodic acid Schiff stains) and search for acid-alcohol-resistant bacilli by the Ziehl-Neelsen staining were negative. A direct fresh examination of lesion scrapings showed no amastigote forms of Leishmaniasis and the chest X-ray was normal, with a 19-mm PPD (purified protein derivative) test (Fig. 2). It was not possible to perform the serum PCR exam due to its unavailability at the time.

**Figure 2:**
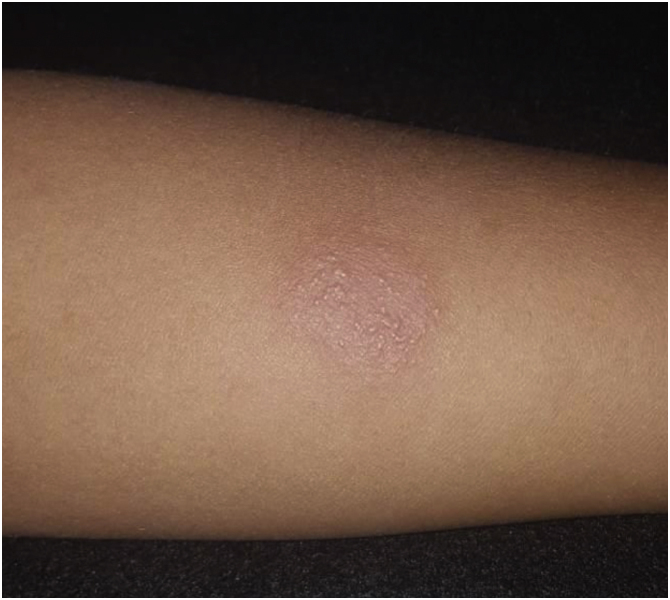
A strongly reactive 19 mm PPD test.

After a trial treatment for tuberculosis (Rifampicin, Isoniazid, Pyrazinamide for two months; Rifampicin and Isoniazid for four months) was initiated, the patient showed significant lesion improvement, with local healing (Fig. 3).

**Figure 3:**
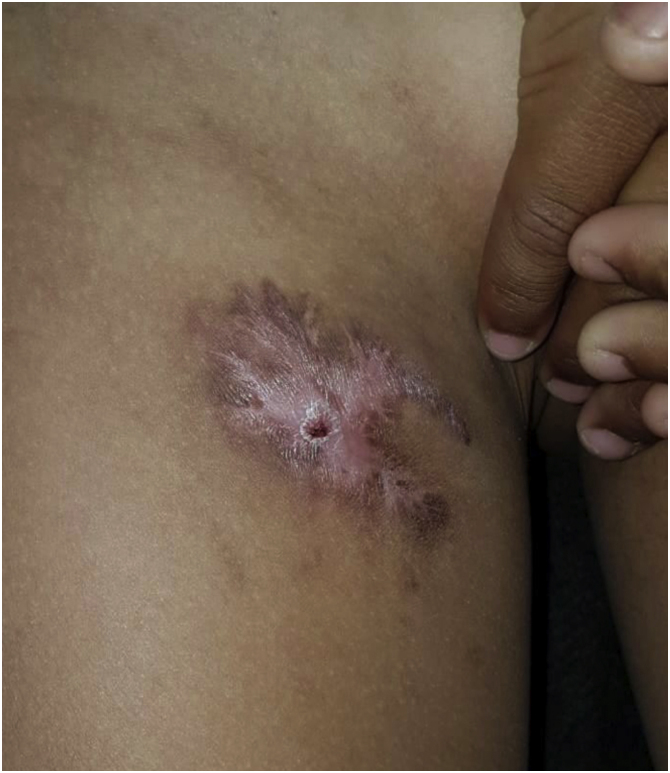
Evolution with significant lesion improvement, after a trial treatment for tuberculosis.

Cutaneous tuberculosis chancre constitutes a rare form of CTB and is equivalent to the primary pulmonary complex. Also called primary inoculation chancre, it develops in people not previously sensitized, occurring more frequently in children. It occurs by direct inoculation into the skin after local, often unnoticed, trauma, with subsequent appearance of an ulcerated inflammatory papule, plaque or nodule, and regional lymphadenopathy.^1^ Histopathology varies according to the time lapse after inoculation: initially, there is a nonspecific acute inflammatory infiltrate. Subsequently, granuloma formation and a decrease in the number of bacilli occur. In most samples, the bacilli will not be observed even with special staining, which makes other complementary tests useful, such as PPD and/or serum PCR, clinical follow-up, and, often, therapeutic testing. When there is clinical suspicion, a histopathological examination with suggestive features, plus a strongly reactive positive PPD, the diagnosis should be suspected. Treatment is similar to that of pulmonary forms of the disease.^4^, ^5^

## Financial support

None declared.

## Authors’ contributions

Gabriela Roncada Haddad: Drafting and editing of the manuscript; effective participation in research orientation; intellectual participation in the propaedeutic and/or therapeutic conduct of the studied cases.

Lívia Caramaschi Florêncio: Drafting and editing of the manuscript; intellectual participation in the propaedeutic and/or therapeutic conduct of the studied cases.

Vidal Haddad Junior: Drafting and editing of the manuscript; effective participation in research orientation; intellectual participation in the propaedeutic and/or therapeutic conduct of the studied cases.

## Conflicts of interest

None declared.
